# Reduced Dislocation of GaAs Layer Grown on Ge-Buffered Si (001) Substrate Using Dislocation Filter Layers for an O-Band InAs/GaAs Quantum Dot Narrow-Ridge Laser

**DOI:** 10.3390/mi13101579

**Published:** 2022-09-22

**Authors:** Yong Du, Wenqi Wei, Buqing Xu, Guilei Wang, Ben Li, Yuanhao Miao, Xuewei Zhao, Zhenzhen Kong, Hongxiao Lin, Jiahan Yu, Jiale Su, Yan Dong, Wenwu Wang, Tianchun Ye, Jianjun Zhang, Henry H. Radamson

**Affiliations:** 1 Institute of Microelectronics, Chinese Academy of Sciences, Beijing 100029, China; 2 Beijing National Laboratory for Condensed Matter Physics, Institute of Physics, Chinese Academy of Sciences, Beijing 100190, China; 3 Beijing Superstring Academy of Memory Technology, Beijing 100176, China; 4 Research and Development Center of Optoelectronic Hybrid IC, Guangdong Greater Bay Area Institute of Integrated Circuit and System, Guangzhou 510535, China

**Keywords:** Si photonics, InAs/GaAs, Quantum dots, III-V epitaxy, dislocation filter layers, defects, superlattice

## Abstract

The development of the low dislocation density of the Si-based GaAs buffer is considered the key technical route for realizing InAs/GaAs quantum dot lasers for photonic integrated circuits. To prepare the high-quality GaAs layer on the Si substrate, we employed an engineered Ge-buffer on Si, used thermal cycle annealing, and introduced filtering layers, e.g., strained-layer superlattices, to control/reduce the threading dislocation density in the active part of the laser. In this way, a low defect density of 2.9 × 10^7^ cm^−2^ could be achieved in the GaAs layer with a surface roughness of 1.01 nm. Transmission electron microscopy has been applied to study the effect of cycling, annealing, and filtering layers for blocking or bending threading-dislocation into the InAs QDs active region of the laser. In addition, the dependence of optical properties of InAs QDs on the growth temperature was also investigated. The results show that a density of 3.4 × 10^10^ InAs quantum dots could be grown at 450 °C, and the photoluminescence exhibits emission wavelengths of 1274 nm with a fullwidth at half-maximum (FWHM) equal to 32 nm at room temperature. The laser structure demonstrates a peak at 1.27 μm with an FWHM equal to 2.6 nm under a continuous-wave operation with a threshold current density of ∼158 A/cm^2^ for a 4-μm narrow-ridge width InAs QD device. This work, therefore, paves the path for a monolithic solution for photonic integrated circuits when III−V light sources (which is required for Si photonics) are grown on a Ge-platform (engineered Ge-buffer on Si) for the integration of the CMOS part with other photonic devices on the same chip in near future.

## 1. Introduction

The Si-based optoelectronic integration chip (OEIC) plays a promising role in cloud-based applications and data centers due to its potential prospects for integrating photonic devices with the mature CMOS technology [1,2,3,4,5]. The OEIC systems mainly consist of numerous independent devices, such as: lasers [6,7], modulators [8,9], detectors [10,11], and waveguides [12], etc. From a practical perspective, Si–based, efficient, and reliable light-emitting sources have long been considered as the “holy grail” of Si photonics due to the many challenges [13]. Unfortunately, Group-IV semiconductors, such as Si [14], Ge [15,16], and GeSi [17,18], which are widely used in integrated circuits, are inefficient light-emitting materials due to their indirect bandgap. Recently, GeSn materials have demonstrated a direct bandgap property, but lasing efficiency at room temperature has still not been demonstrated [19,20,21]. Therefore, low-cost, high-yield, and reliable integrated silicon-based on-chip lasers with emission wavelengths at both 1300 nm (O-band) and 1550 nm (C/L-band) are strongly recommended for realizing the large-scale optoelectronic integration. In contrast, the direct band-gap structure of III–V QD lasers are believed to be the optimal solution for the on-chip integration due to their superior efficiency as a light source [22,23,24]. In particular, self-assembled InAs/GaAs QDs exhibit low-threshold current density, insensitivity to defects, and higher temperature stability, and is widely used as an active optical region for the 1300 nm (O-band) emission spectrum [25,26]. Early studies have reported that direct epitaxial-growth III–V lasers on Si substrates are a promising approach for the cost-effective and high-volume integration of OEICs [27,28].

Nevertheless, the direct epitaxial growth of GaAs materials on Si substrates encounters three major challenges [29]: large lattice mismatch, polarity difference, and thermal expansion mismatch. These problems lead to the formation of threading dislocations (TDs) and antiphase boundaries (APBs), respectively. The TDs that propagate into the active region act as non-radiative carrier recombination centers, reducing minority-carrier lifetimes and, hence, degrade the properties of the photonic device [30,31]. Historically, several attempts were made to avoid APBs, such as using a special offcut substrate [32,33], V-grooved trenches in Si [26,34], and annealing to form double-atom steps on the Si surface. However, even though these methods can eliminate the APB problem, the complexity of the process and the roughness of the GaAs surface cannot meet the requirement for device fabrication and further integration. To reduce the threading dislocation density (TDDs), dislocation filter structures [13,35], a Ge/GeSi buffer layer [36,37], and thermal annealing [38] have been used. Recently, the combined strategy of AlAs nucleation has been demonstrated to grow low TDDs of the GaAs layer on the Si substrate. For instance, Yong-Ho Ko [39] directly grew a low TDD of 2.5 × 10^7^/cm^2^ of the GaAs layer on the silicon (001) substrate using a hybrid model comprising of AlAs nucleation and thermal cycle annealing. Siming Chen [24] grew a low TDD of the 10^5^ cm^−2^ order in the III–V epilayers by combining an AlAs-nucleation layer and In_0.18_Ga_0.82_As/GaAs-dislocation filter layers with in situ thermal annealing, which realized a low-threshold current density of 62.5 A cm^−2^ s for an InAs/GaAs QD laser. Nevertheless, further progress in lowering the TDD (<10^7^ cm^−2^) of high-crystalline quality GaAs/Si substrates to improve the performance of InAs/GaAs QD lasers is urgently needed. In addition, the integration of III-V lasers with other Ge devices, such as Ge modulators [9] and Ge detectors [10], on a single chip is the core goal of the future silicon-based optoelectronic integration.

In this work, we demonstrated a high-quality APB-Free GaAs layer (300 nm) on a Ge buffer grown on 8-inch 6°off-cut Si (001) substrates using reduced-pressure chemical vapor deposition (RPCVD) and metal–organic chemical vapor deposition (MOCVD) tools. The detailed information about the growth has been reported in our previous work [33]. Three sets of strained-layer superlattices (SLSs) were grown as dislocation filter layers (DFLs), and two thermal cycle annealing (TCA) processes were designed to reduce the TDD. Finally, a typical O-band structure with a seven-layer InAs/GaAs dot-in-a-well (DWELL) structure was grown at 420–480 °C. The effect of the growth temperature on the topography and optical properties of InAs QDs was investigated. The structural integrity, defect formation, lattice distortion, surface roughness, and optical performance were characterized using Scanning electron microscopy (SEM), electron-channeling contrast imaging (ECCI), High-resolution transmission electron microscopy (HRTEM), Atomic force microscopy (AFM), High-resolution x-ray diffraction (HR-XRD), and Photoluminescence (PL).

This study introduces novel strained-superlattice structures to filter threading dislocations in the InAs/GaAs QD structure as an active part for the O-band emission. A systematic, optimized GaAs-growth and thermal-cycling annealing of the structure reduced the TDD in the GaAs layer to a low level of ~2.9 × 10^7^ cm^−2^ with a surface roughness of 1 nm. The whole III-V laser structure was grown on an engineered Ge buffer grown on Si. The evolution of the TDD decreased at each stage of the laser structure and was characterized by microscopic HRTEM and ECCL techniques. The laser structures in this work were grown on a Ge buffer/Si substrate, which may provide a monolithic solution for integrating III–V lasers with Si-based photonics and electronics on a single chip in near future.

## 2. Experimental Details

**Figure 1:**
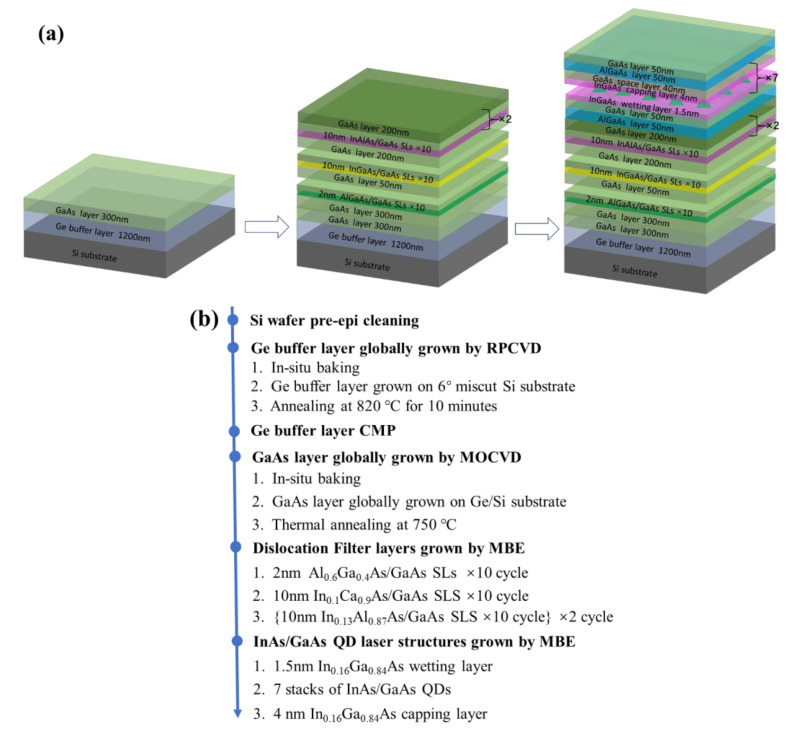
(**a**) Schematic diagram of InAs/GaAs QD grown on Ge-buffered Si substrate with multi-SLSs dislocation filter layers; (**b**) Flow process of InAs/GaAs QD.

The schematic image of the designed structures and their flow process in this work are shown in Figure 1a, b. Seven layers of InAs/GaAs QDs were monolithically grown on three sets of strained-layer superlattices (SLSs) formed on the engineered Ge buffer on 6° off-cut (001) Si substrates. First, a standard cleaning procedure for Si substrates prior to epitaxy were published in [40,41]. Initially, a Ge buffer layer of 1400 nm was grown on 6° off-cut Si (001) 200 mm wafers in an RPCVD ASM Epsilon 2000 reactor (ASM Inc., Almere, The Netherlands). The Ge buffer growth was performed at a low temperature of 450 °C followed by a high temperature of 650 °C. The first layer had a high defect density due to the nucleation of Ge on Si, while the second layer had a remarkably lower TDD. The Ge buffer underwent thermal annealing at 850 °C to improve the epitaxial quality. A Chemical Mechanical Polish (CMP) was applied to ensure the surface roughness remained below 1 nm. Later, a 300 nm-GaAs layer was grown, followed by three types of superlattices (Al_0.6_Ga_0.4_As/GaAs, In_0.1_Ga_0.9_As/GaAs, and In_0.13_Al_0.87_As/GaAs) and 7 periods of InAs/GaAs DWELLs, each separated by a 40-nm GaAs spacer layer structure, shown in Figure 1a.

The GaAs growth was performed In three steps in a commercial, low-pressure metalorganic chemical vapor deposition Reactor (AIXTRON 701900 MOCVD equipment, Germany). The GaAs layer consisted of an 18 nm GaAs nucleation layer at 460 °C, a 120 nm middle-temperature GaAs buffer layer at 600 °C, and a 160 nm high-temperature GaAs layer at 680 °C. To reduce the dislocation density of the GaAs layer, the structures were subjected to cyclic annealing. One annealing cycle involved a period of 750 °C-high-temperature annealing for 5 min following the 160 nm high-temperature GaAs layer, then reducing the temperature to 350 °C, after which this TCA process was repeated five times. The second annealing process involved the growth of the HT-GaAs layer to 80 nm at 680 °C, followed by the one-step five-cycle TCA; then the second group of HT GaAs was grown to another 80 nm at 680 °C, followed by a second round of the TCA. Each set of the TCA was performed between 350 °C and 750 °C under an arsine and hydrogen ambient.

Three sets of dislocation filter layers (DFLs) were deposited on the GaAs/Ge/Si (001) substrate using a solid-source molecular beam epitaxy (MBE). To suppress the propagation of TDs, a 300 nm thick GaAs main layer and ten periods of Al_0.6_Ga_0.4_As (2 nm)/GaAs (2 nm) superlattices (SLs) were both first grown at 580 °C. In order to further reduce the TDD and flatten the surface, one-repeat In_0.1_Ga_0.9_As/GaAs and two-repeat In_0.13_Al_0.87_As/GaAs SLs structures were deposited at 470 °C as DFLs. All the DFLs consisted of ten periods of 10 nm InGa(Al)As strained layers and 10 nm GaAs layers, which were separated by the 200 nm GaAs main layers. Finally, another 100 nm thick GaAs layer was grown at 580 °C with a growth rate of 0.6 Å/s to further smoothen the surface of the sample.

The active InAs/GaAs QD layers were subsequently grown on such GaAs/Ge/Si (001) substrates. First, a standard seven-layer InAs/GaAs DWELL structure was deposited on the GaAs/Ge/Si substrate at 420–480 °C. Each of the DWELL layers included a 3 ML InAs QD layer, which was sandwiched by a 1.5 nm In_0.16_Ga_0.84_As wetting layer and a 4 nm In_0.16_Ga_0.84_As capping layer, all grown at 450 °C. All the DWELLs were separated by 40 nm GaAs spacer layers. Moreover, to increase the quantum efficiency, two layers of 50 nm Al_0.35_Ga_0.65_As film were grown above and below the DWELL active region as the carrier confinement layers. Lastly, surface InAs QDs were deposited on the structure with the same growth conditions as the buried ones, for AFM characterization.

## 3. Results and Discussion

### 3.1. Thermal Cycle Annealing (TCA) Investigation

**Figure 2:**
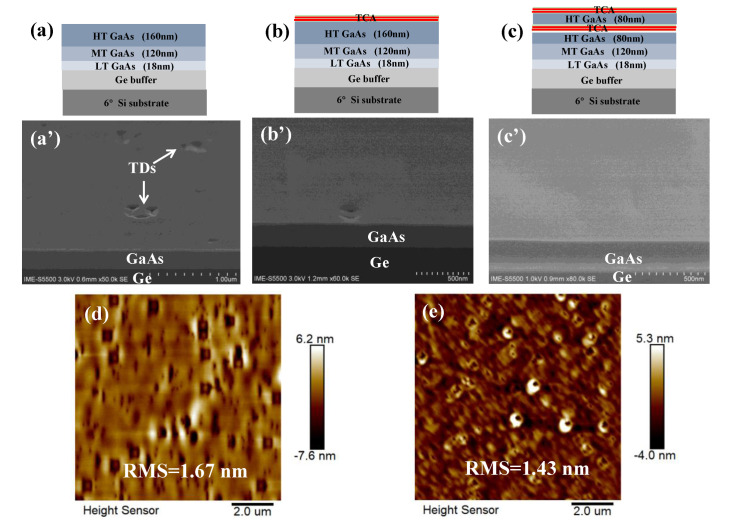
Schematic of the layered structure of the third step of the GaAs/Ge/Si template grown (**a**) without the TCA step; (**b**) with TCA steps in 0.16 μm-thick HT GaAs; (**c**) with TCA steps in an initially 0.08 μm-thick substrate followed by continuous TCA of the topmost surface of the template. (**a′**–**c′**) SEM images of the GaAs surface for the processes of (**a**–**c**), while (**d**,**e**) are AFM images of samples (**a**,**c**) (10 × 10 µm^2^ area scan).

The evolution of the defect density of the epi-layers was studied stepwise, starting with the GaAs epitaxy, which is the first III-V layer on the Ge buffer/Si substrate, and the effect of TCA on the epitaxial quality. The GaAs growth was optimized by l three-step growth process, and the first GaAs layer in Figure 2a is without post-annealing (as a reference sample). The second GaAs in Figure 2b underwent five TCA steps following the 160 nm HT-GaAs layer. The third GaAs layer in Figure 2c underwent five TCA steps at the initial HT-GaAs of 80 nm, then five continuous TCA steps were continuously performed at the topmost 80 nm HT-GaAs layers. Then, the effect of annealing processes on the GaAs surface morphology was characterized using SEM, as demonstrated in Figure 2a′–c′.

The GaAs layer grown without TCA in Figure 2a′ illustrates a high TDD, under which these defects were generated during epitaxy and propagated towards the surface. The surface morphology of this sample is shown in the AFM image in Figure 2d. Many small defects were observed, which exhibited rough surfaces on the GaAs layer with an RMS of 1.67 nm. In comparison, a one-step TCA process was carried out after the growth of a 160 nm HT-GaAs layer and the void defect density on the GaAs surface remarkably decreased (in Figure 2b′), exhibiting flatter surfaces compared to the samples without TCA. On the other hand, in the case of the two-step cyclic annealing process (Figure 2c), a few vague pits can be observed on the GaAs surface from the SEM image in Figure 2c′, indicating threading dislocations were significantly reduced. This GaAs growth after each cycle annealing shows a further decrease of defects, and this occurs not only for the newly deposited GaAs part but also for the entire GaAs layer. AFM characterization also revealed that the surface roughness of GaAs decreased to 1.43 nm. The mechanism of defect reduction of the GaAs film using the TCA process is based on the thermal stress forcing the existing TDs to glide during TCA, after which, the glided TDs may react with existing TDs and fail to propagate through the epi-layer.

### 3.2. Dislocation Filter Layers (DFL) Efficiency

**Figure 3:**
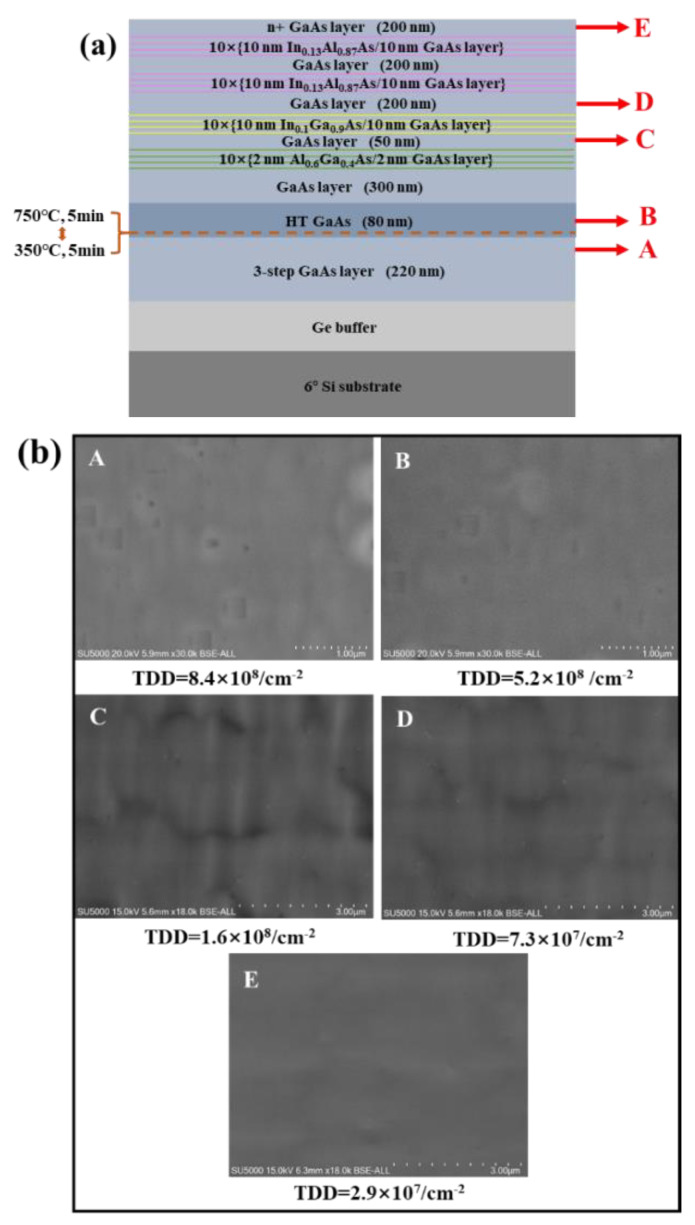
(**a**) Schematic diagram of the complete, optimized GaAs/Ge/Si structure; (**b**) ECCI images at different growth stages, labeled as “A, B, C, D and E”.

Although there are several reports using the DFL method and demonstrating a relatively flat GaAs surface, the defect density does not meet the requirement for a high performance of InAs QDs lasers monolithically grown on the Si substrate. The remarkable extension of the QD device’s lifetime requires a lower dislocation density GaAs/Ge/Si platform; therefore, there is an urgent need for developing better-quality GaAs buffers. SLSs are widely applied as dislocation filter layers to effectively reduce TDD that is due to alternating signs of misfit strain and to enhance the probability for dislocation interactions as the threading segments move back and forth [42,43,44]. In this work, we studied three different types of SLSs: Al_0.6_Ga_0.4_As/GaAs SLSs, In_0.1_Ga_0.9_As/GaAs SLSs, and In_0.13_Al_0.87_As/GaAs. Figure 3a illustrates the schematic of the growth structure, along with three sets of SLSs. To compare the efficiency of different dislocation filtering approaches, we terminated the GaAs-buffered growth at various stages (labeled A, B, C, D, and E) and subjected the samples to an ECCI analysis. Compared with the etch pits density (EPD) method to measure TD density, the ECCI has been reported to be a more effective technique for estimating the TDD in GaAs since it is a non-destructive measurement. The ECCI images were shown in Figure 3b where the dislocations were visualized as brightly contrasting marks in the ECCI image.

According to the quantitative ECCI scans in Figure 3b, the dislocation density was decreased by 38% after TCA (from 8.4 × 10^8^ in the labeled A area to 5.2 × 10^8^ cm^−2^ in the labeled B area). First, we identified the ten-period 2 nm Al_0.6_Ga_0.4_As/2nm GaAs SLSs as DFLs to block the propagation of threading dislocations from the bottom GaAs layer. As seen from Figure 3b, the labeled C area has a TDD of 1.6 × 10^8^ cm^−2^, which is significantly lower than that of the labeled B area. Then, the next ten-period 10 nm In_0.1_Ga_0.9_As/10 nm GaAs superlattices were inserted to further filter the dislocations labeled D. A remarkable reduction of dislocations to a 10^7^ cm^−2^ magnitude was observed. However, it should be noted that, although a considerable number of TDs were effectively blocked, some small pits still existed at the topmost layer of the GaAs surface. Meanwhile, with the additional growth of two cycles of the ten-period of the 10 nm In_0.13_Al_0.87_As/10 nm GaAs SLs structures, a smooth GaAs surface was observed, as shown in Figure 3b, in the labeled E area. A TDD of 2.9 × 10^7^ cm^−2^ was measured via the ECCI method, which is two orders of magnitude lower than that of the previous case of inserted DFLs. In conclusion, inserting a superlattice dislocation filter layer is effective at reducing the Si-based GaAs dislocation density. In our work, we obtained a low TDD of 2.9 × 10^7^ cm^−2^ for a 1.8 μm GaAs layer.

**Figure 4:**
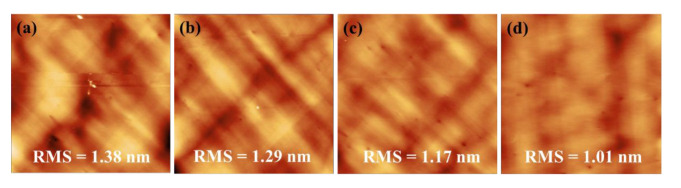
AFM image of the GaAs buffer at different growth stages as marked in Figure 3a as: (**a**) labeled B; (**b**) labeled C; (**c**) labeled D; and (**d**) labeled E. The scans were performed for 10 × 10 μm^2^ areas on the sample.

To gain further insight into the effects of DFLs, AFM scanning was performed on the surface of the GaAs-buffered growth at various stages, labeled B, C, D, and E (in the Schematic diagram of Figure 3a) as shown in Figure 4. For GaAs epitaxial films grown on a Ge/Si substrate without DFLs, relatively rough surfaces were observed with an RMS roughness of 1.38 nm, but several pits were visible in Figure 4a. After depositing three types of strained superlattice layers, the roughness was reduced from 1.29 nm and 1.17 nm to 1.01 nm. In Figure 4b–d, the pits on the GaAs surface were decreased to almost none. The mechanism behind the successive improvement is attributable to the SLS dislocation filter layers. These SLSs not only filter the threading dislocation, which could otherwise extend to the GaAs surface, but also work as an energy absorption region to ease the thermal strain caused by the coefficient of thermal expansion between the Si and GaAs.

**Figure 5:**
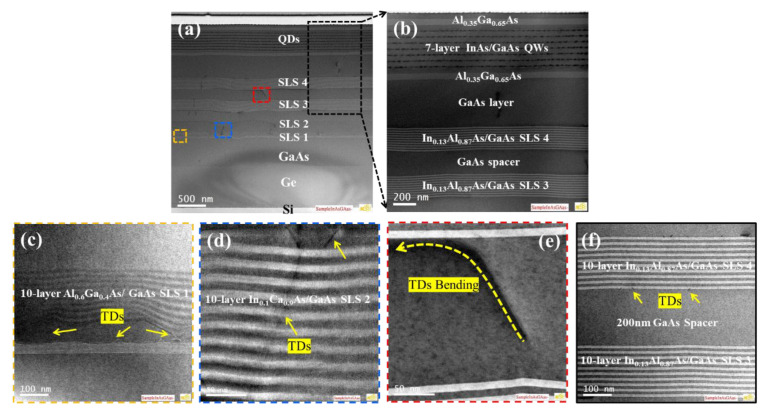
(**a**) Cross-sectional bright-field STEM image of seven-layer InAs QDs on the GaAs/Ge/Si substrate inserted with SLS; (**b**) close-up view of the QD region; (**c**) magnified image of the first Al_0.6_Ga_0.4_As/GaAs SLS; (**d**) magnified image of the second In_0.1_Ga_0.9_As/GaAs SLS; (**e**) Zoomed-in cross-sectional TEM images of TDs bending at the SLS interface; (**f**) Higher resolution STEM image that demonstrates the sharp interface between In_0.13_As_0.87_As and GaAs.

Based on the above macroscopic analysis, we may conclude that the SLS has a direct and significant impact on improving the quality of the GaAs film. In addition, cross-sectional TEM analysis has been performed to examine the crystal quality. Figure 5a (and Figure 5b is the marked area) presents the bright-field (BF) STEM image of the complete seven-layer InAs QDs grown on the Ge/Si substrate inserted with four stages of the SLS dislocation filter layer. It is clearly visible in the enlarged marked area in Figure 5b that the GaAs layer, after the fourth set of SLSs, has minor TDD, providing a good platform for the growth of high-quality InAs/GaAs QWs. Herein, to directly monitor the generation and propagation of the defects in detail, zoomed-in STEM images are shown in Figure 5c–f. For the first set of 2 nm Al_0.6_Ga_0.4_As/2 nm GaAs SLS insertions, the penetrating dislocation defects were mostly bent over at the bottom or top interfaces of each set of SLSs, and a few dislocations were injected into the upward GaAs epilayer, as shown in Figure 5c. After the first set of SLSs, the dislocation density was reduced by over one order of magnitude (10^8^ cm^−2^). However, it was also noted that some threading dislocations were confined or coalesced to form line defects, which could propagate upward to the second 10 nm In_0.1_Ga_0.9_As/GaAs SLS and be terminated at the top surface according to the TEM image in Figure 5d. In addition to the sharp interfaces of the SLSs, apparent TD bending and termination at the InGaAs/GaAs hetero-interfaces were observed, as indicated by the yellow arrows shown in Figure 5e. Figure 5f shows the on-axis TEM image used to assess the interface roughness of the In_0.13_Al_0.87_As/GaAs SLSs. It was noted that arrays of misfit dislocations (MDs) were pinned at the interfaces between the SLSs and the GaAs spacer. The emergence of in-plane MDs indicated that the strain field was sufficient to affect the dislocation movement. The defect density is estimated at 8 × 10^6^ cm^−2^ after the fourth set of In_0.13_Al_0.87_As/GaAs SLSs.

**Figure 6:**
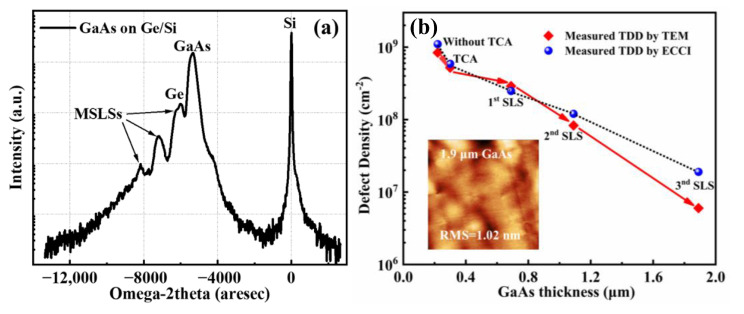
(**a**) HRXRD (004) RCs of GaAs layers on Ge-on-Si substrates with multi-SLS layers; (**b**) Extracted dislocation density as a function of the GaAs buffer thickness at various growth stages. The inset shows the 10 × 10 μm^2^ AFM scan of the 1.9 μm GaAs buffer inserted with SLSs.

In order to further analyze the material quality, the HRXRD rocking curve (004) of the GaAs layer on the Ge-on-Si substrate was characterized in Figure 6a. The high-intensity peak and narrow broadening of GaAs and Ge peaks in the rocking curve indicate a high crystalline quality of the GaAs layer. In the rocking curve, the multiple peaks from the Al_0.6_Ga_0.4_As/GaAs, In_0.1_Ga_0.9_As/GaAs, and In_0.13_As_0.87_As/GaAs SLSs were also distinguished, which confirmed the high quality of the III-V buffer layer. In addition, the evolution of the dislocation density with different GaAs thicknesses and those undergoing various filtering approaches are summarized in Figure 6b. ECCI and TEM measurements were taken to quantify the dislocation density of GaAs after inserting each DFL structure. As can be seen from the plot, the dislocation density of the GaAs buffer was reduced from ∼10^9^ cm^−2^ to ∼10^7^ cm^−2^ after inserting various stages of SLS.

After the first and second sets of SLS, the two characterization techniques had similar results for estimating the TDD. However, after inserting the third SLS, the estimations of the TDD results differed as follows: 2.9 × 10^7^ cm^−2^ and 8 × 10^6^ cm^−2^ were obtained from ECCI and TEM analysis, respectively. The TEM measurement became unreliable for quantitative comparison. This discrepancy occurs when the TDD is lower than 10^7^ cm^−2^ in magnitude when the most of the defects have disappeared and rose to the surface.

To examine the efficiency of different types of SLS, an ECCI analysis is used for measuring dislocation density just above each layer of SLS. The dependence of the dislocation density $\;\rho_{TD}$) on the GaAs thickness *d* can be experimentally fitted by a power law [45]:

(1) $$\rho_{TD}=10^{8.93}\cdot d^{-0.963}$$

Here, the efficiencies of three sets of SLSs are 54%, 86%, and 92%, respectively. This was observed when the SLSs were optimized, and the strain field provided by the SLS was enough to drive the adjacent TDs into coalescence, which enhances the lateral motion of TDs considerably, and hence, increases the probability of annihilation.

### 3.3. Growth of InAs QDs

**Figure 7:**
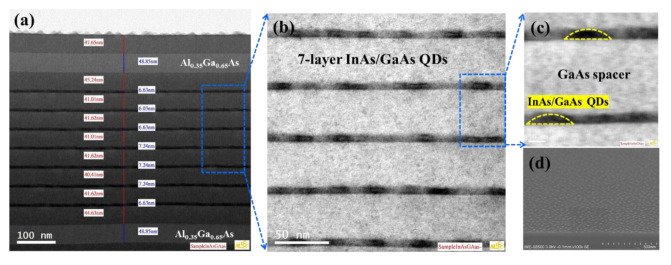
(**a**) Cross-sectional dark field HRTEM image of a defect-free seven-layer QD active region with Al_0.35_Ga_0.65_As cladding on both sides grown at 450 °C; (**b**) magnified image of InAs QDs; (**c**) Zoomed-in morphology of InAs QDs with a diameter of ~24 nm and height of ~7 nm; (**d**) Tilted-SEM image of the InAs QDs surface.

Figure 7 shows the TEM images of a seven-layer InAs QDs active region grown at 450°C with Al_0.35_Ga_0.65_As cladding on both sides, grown on the GaAs/Ge/Si substrate after DFL insertion. As is shown in Figure 7a, each layer in the active region is smooth and contains nearly no defects throughout the whole structure, indicating the high quality of the GaAs buffer layers. In addition, the ~41 nm GaAs space and ~7 nm InAs QDs were in accordance with our design requirement. From the magnified image of active region, the profile of each stack of InAs/GaAs was clearly distinguishable and evenly distributed, and the overall quality of the structure exhibited good quality. The zoomed-in TEM image of the InAs QDs in Figure 7c depicts a regular hemispherical shape with uniform distributions. The typical dot size was ∼24 nm in diameter and ∼7 nm in height with an aspect ratio of ∼0.29. Furthermore, the morphology of SEM images further verified the hemispherical shape and uniform distribution of InAs QDs from Figure 7d.

**Figure 8:**
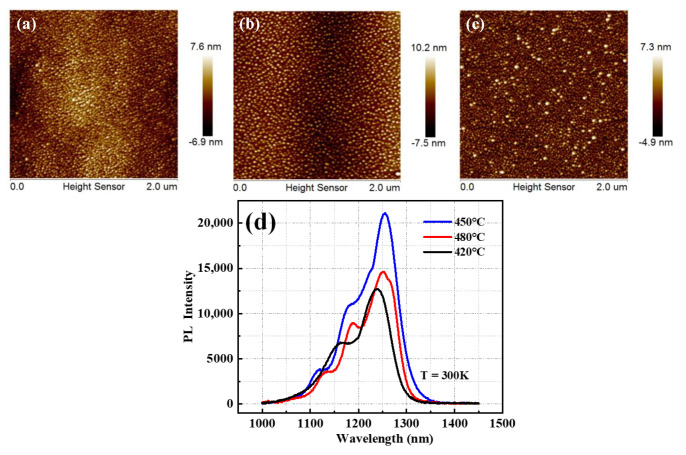
(**a**–**c**) AFM images of InAs QDs grown on the GaAs/Ge/Si substrate at 0.1 ML/s with the same deposition thickness of 3.1 ML but different temperatures: 420 °C, 450 °C, and 480 °C, in sequence; and (**d**) room-temperature photoluminescent spectra of InAs/GaAs QDs grown at the aforementioned growth t temperatures.

Figure 8 displays the influence of temperature on the morphology of InAs QDs via AFM images and the photoluminescence (PL) structure. The AFM images revealed that when the temperature is raised, the density of dots decreased. For example, at growth temperatures, 420 °C, 450 °C, and 480 °C, the InAs QDs densities are 4.2 × 10^10^ cm^−2^, 3.4 × 10^10^ cm^−2^, and 2.6 × 10^10^ cm^−2^, in sequence. It was also noted that the uniformity was greatly affected and the QD size became larger at a higher temperature. This result is mainly attributed to the low migration and diffusion length of an In adatom at a low growth temperature.

A PL analysis at room temperature was performed to evaluate the optical properties of QDs grown at different temperatures, as shown in Figure 8d. In these measurements, a laser (HORIBA iHR550) with a 532 nm wavelength for continuous-wave optical pumping and a liquid nitrogen-cooled InGaAs linear array detector was applied. The results showed that when the growth temperature was increased from 420 °C to 450 °C, the wavelength and the intensity of PL spectra are increased; meanwhile, for the sample grown at 480 °C, the PL intensity strongly decreased. We observed that InAs/GaAs QDs grown at 450 °C had the best PL intensities among the samples, exhibiting a 1274 nm emission wavelength with a narrower PL emission bandwidth of only 32 nm, which is attributable to the high quality of InAs QDs. Theoretically, the high density of quantum dots grown at a low temperature have a stronger PL peak intensity, but in reality, In atoms deposited at low temperatures are more likely to form giant quantum dots containing defects, which can also offset the material optical quality leading the PL intensity reduction. In addition, the numeral peak of PL spectra also verifies the performance of InAs QDs. For InAs/GaAs QDs grown at 450 °C, two peak positions were set at 1125 nm and 1274 nm, which were associated with the ground-state and excited-state emissions in the O-band of the telecommunication window. However, a multiple-peak PL spectrum was observed for the samples grown at 420 °C and 480 °C, which could be attributed to the non-uniformity of QD sizes. The mechanism behind this could be explained by the optical properties of InAs QDs predominately depending on the formation of strain-induced defects with increasing QD sizes. The S-K growth model of 3D InAs QDs grown on the GaAs substrate was dominant, and it takes advantage of the strain energy generated by the lattice mismatch between materials. The growth temperature had a great influence on the strain relaxation of 3D InAs quantum dots, which affect the bandgap of InAs QDs, showing the red-shift of the wave peak in the PL spectrum. This conclusion also relates to Varshni’s law [46].

### 3.4. Device Fabrication

**Figure 9:**
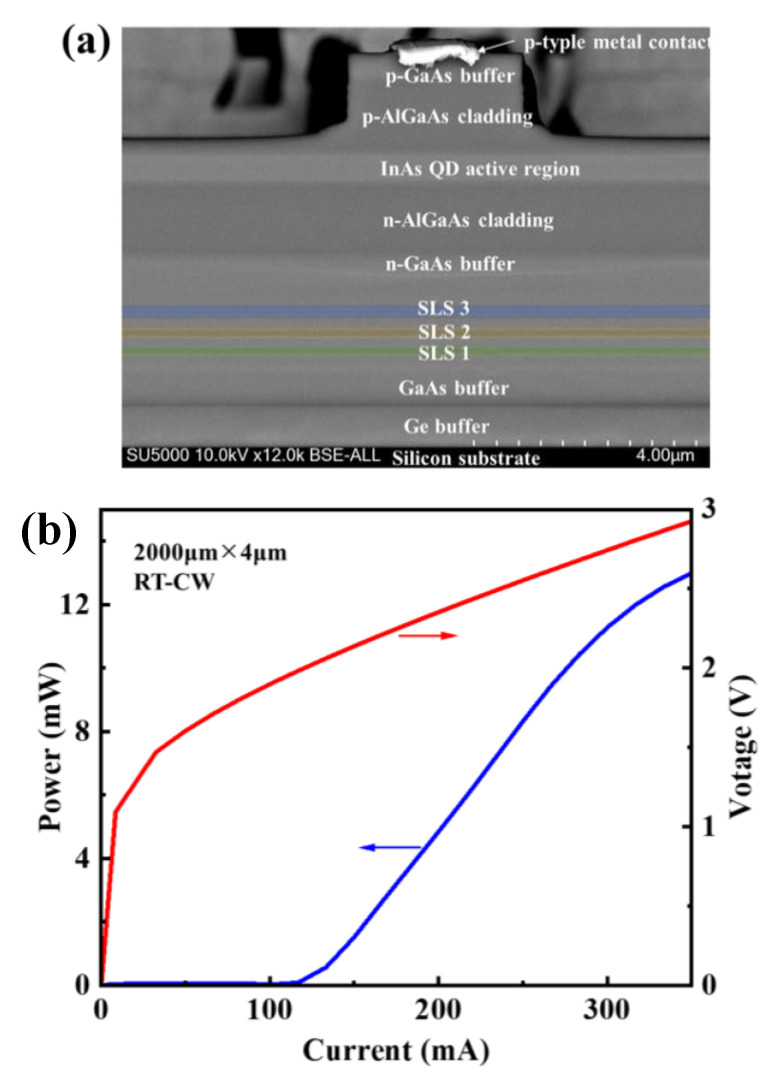

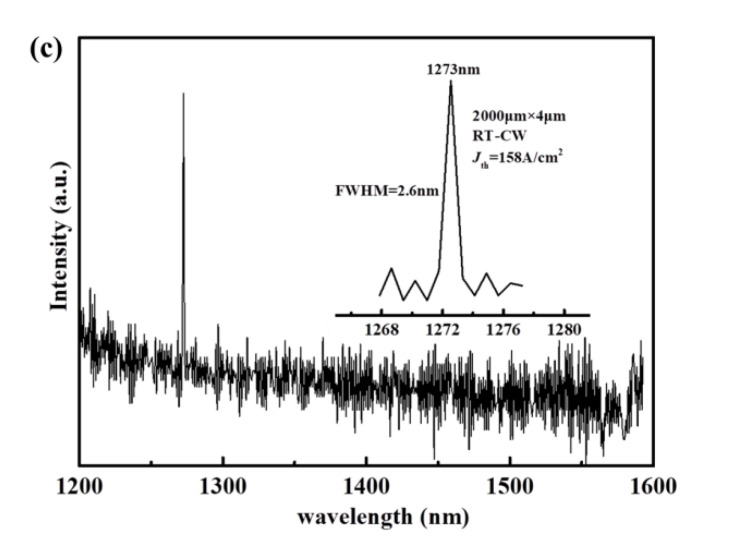
(**a**) Cross-sectional SEM image of the InAs/GaAs QD laser structure after dicing and with the narrow-ridge width of 4 µm; (**b**) L–I–V curve of InAs QD lasers under CW mode at RT; and (**c**) Emission spectrum of InAs QD laser at RT and CW modes. The insert of (**c**) illustrates the magnification of the peak position.

On the basis of the optimized conditions, an InAs/GaAs QD Fabry–Perot (FP) laser structure was developed on the prepared GaAs/Ge/Si substrate. A p-doped upper cladding layer and n-doped lower cladding layers of 1.4 μm Al_0.4_Ga_0.6_As were used to confine the seven-layer DWELL structures. A 300 nm p-doped GaAs contact layer completed the growth of the laser structure. The n- and p-contacts were formed by depositing NiGeAu/Au and Ti/Pt/Ti/Au on the exposed n-doped GaAs contact layer and top p-contact layer [47], respectively. Subsequently, rapid post-annealing was performed at 385 °C (n-contact) and 425 °C (p-contact) for conforming the ohmic contact between metal and semiconductors. Then, process steps were as follows: standard lithography, inductively coupled plasma (ICP) etching, SiO_2_ deposition, reactive ion etching (RIE), and electrode evaporation were fabricated as in [48]. The cross-sectional of the laser structure is shown in the SEM image in Figure 9a.

Figure 9b shows the light-current-voltage (LIV) characteristic measurements (DR-BAR-AT300) of the InAs/GaAs QD laser with a 4μm narrow-ridge width and a 2000 μm cavity length based on and under a continuous wave (CW) model operation at room temperature (RT). The red solid line represents the IV curve of the CW mode, which indicates good electrical contacts with the laser diode. The blue solid line represents the LI curve of the CW mode at RT condition and the lowest threshold current (*I_th_*) of 118mA, which represents a threshold current density (*J_th_*) of 146 A/cm^2^. The maximum output power (Pout) of the laser was 12 mW. Figure 9c shows the emission spectra of the InAs/GaAs QD laser, which is measured at an injection current density of 158A/cm^−2^ at RT under the CW mode. The emission wavelength from the ground state was 1273 nm, located in the O-band with a line-width (FWHM) of only 2.6 nm (as seen in the inset of Figure 9c) and a sharp intensity, which could be adjusted by tuning the growth parameters of InAs QDs in the active region. It should be noted that the capability of this narrow ridge of InAs QD devices with high performance has promise in its potential application in silicon photonics integrated circuit (PIC) chips with small-sized CMOS devices.

## 4. Conclusions

We have demonstrated a high-performance of InAs/GaAs QD narrow-ridge lasers on Ge/Si substrates with optimized high-quality GaAs buffer layers. Strategies of TCA process and SLSs insertions were investigated to improve the quality of the GaAs buffer layer. Results showed that a remarkable reduction of the TDD to 2.9 × 10^7^ cm^−2^ with a surface roughness of 1.01 nm for a 1.9 μm-thick GaAs “virtual” layer was obtained. On top of the optimized GaAs layer, a high density of 3.4 × 10^10^ cm^−2^ InAs/GaAs QDs grown at 450 °C exhibited a peak wavelength of 1274 nm with an FWHM equal to 32 nm in the RT-PL measurement. In addition, the growth temperature played a key role in the size distribution during the QD formation and in the PL characteristics. The fabricated QD laser device was successfully operated at room temperature. The reduced TDD of the GaAs buffer and the high quality InAs QD region led to a threshold current density of ∼146A/cm^2^ and an output power of 12 mW for a 4 µm × 1 mm ridge laser under an RT-CW model operation. This work demonstrated highly efficient light sources for photonics, but also offers the possibility of the integration of Si-based optoelectronic with advanced Ge-CMOS devices.
